# Extended Adjuvant Tamoxifen for Early Breast Cancer: A Meta-Analysis

**DOI:** 10.1371/journal.pone.0088238

**Published:** 2014-02-20

**Authors:** Mustafa Al-Mubarak, Ariadna Tibau, Arnoud J. Templeton, David W. Cescon, Alberto Ocana, Bostjan Seruga, Eitan Amir

**Affiliations:** 1 Division of Medical Oncology and Hematology, Princess Margaret Cancer Centre and Department of Medicine, University of Toronto, Toronto, Canada; 2 Ministry of Higher Education, Riyadh, Saudi Arabia; 3 Medical Oncology Department and Translational Research Unit, Albacete University Hospital, Albacete, Spain; 4 Sector of Medical Oncology, Institute of Oncology Ljubljana, Ljubljana, Slovenia; Dartmouth, United States of America

## Abstract

**Background:**

Hormone receptor positive breast cancer is characterized by the potential for disease recurrence many years after initial diagnosis. Endocrine therapy has been shown to reduce the risk of such recurrence, but the optimal duration of endocrine therapy remains unclear.

**Methods:**

We conducted a systematic review and meta-analysis to quantify the benefits and harms of extended adjuvant tamoxifen (>5 years of therapy) compared with adjuvant tamoxifen (5 years of therapy). Odds ratios (ORs) and 95% confidence intervals (CIs) were computed for disease recurrence, death and adverse events. Subgroup analyses by timing of recurrence and baseline lymph node and menopause status were carried.

**Results:**

Five trials comprising 21,554 patients were included. Extended adjuvant tamoxifen was not associated with a significant reduction in the risk of recurrence (OR:0.89, 95% CI 0.76–1.05, p = 0.17). There was no association between extended adjuvant tamoxifen and all-cause death (OR:0.99, 95% CI 0.84–1.16, p = 0.88). There was an apparent reduction in risk of recurrence occurring after completion of extended adjuvant tamoxifen with little evidence of effect during therapy, however, this difference was not significant (p for difference 0.10). Subgroup analysis suggested that a greater effect size among lymph node positive patients compared with those who are lymph node negative (NNT: 25 vs. 49). There was no apparent difference in the effect between pre- and post-menopausal patients. Endometrial carcinoma was substantially more frequent with extended adjuvant tamoxifen (OR:2.06, 95% CI 1.65–2.58, p<0.001, number needed to harm:89).

**Conclusion:**

In unselected patients, extended adjuvant tamoxifen is not associated with a significant reduction in recurrence, or a reduction in all-cause death. Patients with lymph node positive breast cancer may derive some benefit. Reduction in the risk of recurrence appears to occur only after completion of extended adjuvant therapy.

## Introduction

Early hormone receptor expressing breast cancers are characterized by the propensity for late recurrence with approximately 50% of recurrences and deaths occurring 10 years or more after completion of 5 years of adjuvant endocrine therapy [1]. Methods for reducing late recurrences such as extended adjuvant hormone therapy are therefore of interest.

Both tamoxifen and aromatase inhibitors (AI) have been studied in the extended adjuvant setting. The use of AI after 5 years of adjuvant tamoxifen has shown a reduction in the risk of breast cancer recurrence in three studies [2]–[4]. In the National Cancer Institute of Canada Clinical Trials Group (NCIC CTG) MA.17 study; treatment with 5 years of letrozole after 4.5–6 years of tamoxifen was associated with improvement in disease free survival (DFS) and distant DFS. However, despite improvements in DFS in both lymph node positive and negative patients, an improvement in overall survival was only seen in patients with node positive disease [2].

Studies of extended adjuvant tamoxifen (more than 5 years of therapy) compared with adjuvant tamoxifen (5 years or less of therapy) have shown mixed results. Two studies, the National Surgical Adjuvant Breast and Bowel Project (NSABP) B-14 [5] and the Scottish adjuvant tamoxifen trial [6] demonstrated no benefit of extended adjuvant tamoxifen. Moreover, the NSABP B-14 study showed in its interim analysis a greater number of recurrence events in those patients treated with extended adjuvant tamoxifen. A number of other studies have shown a reduction in breast cancer recurrence, including the recent results of the aTTom (Adjuvant Tamoxifen–To Offer More?) [7] and ATLAS (Adjuvant Tamoxifen: Longer Against Shorter) trials [8], as well as the combined analysis of the Eastern Cooperative Oncology Group (ECOG) E4181 and E5181 trials [9]; and a French trial [10] which compared 2 or 3 years of adjuvant tamoxifen with long term therapy.

In view of the variability in the results of trials of extended adjuvant tamoxifen, the true benefit of this treatment strategy is unknown. Furthermore, it is unclear if certain subgroups experience differential benefit from extended adjuvant tamoxifen. Here, we report a meta-analysis of randomized trials of extended adjuvant tamoxifen. The objectives were to assess the relative and absolute differences in efficacy and toxicity of extended adjuvant tamoxifen compared with adjuvant tamoxifen. We hypothesized that benefit of extended therapy may be limited to patients with lymph node positive disease, as has been observed with AIs.

## Materials and Methods

### Search Strategy

This study was conducted in accordance with the Preferred Reporting Items for Systematic Reviews and Meta-Analyses [11]. Eligible trials were identified using a computerized search of the following databases: MEDLINE (host: PubMed); EMBASE (host: OVID), 1980–2013 week 20; American Society of Clinical Oncology Annual Meetings, 2005–2013; European Society of Medical Oncology/European Cancer Organization Meetings, 2005–2013; San Antonio Breast Cancer Symposium Annual Meetings, 2005–2012 and the Cochrane Central Register of Controlled Trials from January 1980 to April 2013. The search was restricted to English language articles. The terms “adjuvant,” and “tamoxifen” and “breast cancer” and similar terms were cross-searched by using the following search algorithm: ((tamoxifen) AND (adjuvant) AND(Breast neoplasm MeSH OR ((breast OR mammary) AND (carcinoma OR malignan* OR neoplasm OR tumor)))) AND (randomized controlled trial [pt]OR controlled clinical trial [pt]OR randomized controlled trial [mh]OR double-blind method [mh] OR single-blind method [mh] OR clinical trial [pt] OR clinical trials [mh] OR (“clinical trial”) [tw] OR singl* [tw] OR doubl* [tw] OR trebl* [tw] OR tripl* [tw] AND (ask* [tw] OR blind* [tw])) OR comparative study [mh] OR evaluation studies [mh] OR follow-up studies [mh] OR prospective studies [mh] OR control* [tw] OR prospective* [tw] OR volunteer* [tw] NOT (animals [mh] NOT humans [mh]). Included studies were randomized clinical trials that compared short duration of adjuvant tamoxifen therapy (defined as five years, adjuvant tamoxifen henceforth) to longer adjuvant tamoxifen therapy (defined as more than 5 years, extended adjuvant tamoxifen henceforth) as treatment in women with early breast cancer. Reference lists of eligible studies were also consulted for other relevant articles or abstracts. Both data from published articles and abstracts presented at annual meetings were included in a meta-analysis. The study was not registered and a protocol of the study was not published online.

### Data Extraction

Data on efficacy, safety and tolerability were collected. Efficacy outcomes included risk of recurrence (including contralateral breast cancer), risk of distant recurrence, death without recurrence and death from any cause. Safety and tolerability data included pre-specified adverse events and discontinuation of treatment (defined as failure to continue allocated therapy for duration of trial). The following adverse events were pre-specified in this analysis: cardiac (including myocardial infarction, angina and congestive cardiac failure), venous thromboembolism, endometrial carcinoma and other non-breast malignancies. Events of interest were extracted from the primary publications and from any associated online appendices by two authors (MA-M and EA) using a standardized data extraction form. Discrepancies were resolved by consensus.

### Data Synthesis

The analysis was performed in multiple cohorts. Initial analyses were carried out for data from all randomized patients. Subsequent analyses excluded data from patients whose tumors were shown not to express the estrogen receptor (ER, i.e. a combination of patients who were ER-positive or ER-unknown). A sensitivity analysis was conducted to assess the effect of patients with ER-unknown tumors on estimates of interest. Subgroup analyses were also planned to assess the events of interest in subgroups frequently reported by individual studies. These included assessment by time of recurrence (during extended adjuvant therapy or beyond), lymph node status (negative versus positive) and by menopausal status (premenopausal versus postmenopausal at the time of enrolment).

### Statistical Analysis

Data on relative event rates of interest were expressed as odds ratios (OR) and their respective 95% confidence intervals (CI) for the experimental arms compared with the control groups. Data were then included in a meta-analysis using RevMan 5.1 software (Cochrane Collaboration, Copenhagen, Denmark). Pooled estimates of OR were computed using the Mantel-Haenszel OR method [12], [13]. The random effect model was used if the p-value for Cochran’s Q was <0.1 or if the I^2^ index was >50% [13]. Otherwise, the fixed effect model was utilized. Differences between the subgroups were assessed using methods described by Deeks et al [14]. Absolute risks of event of interest were calculated as the number of events per person after 10 years of follow-up where available. The difference in absolute risk between extended adjuvant and adjuvant tamoxifen was also presented as the number needed to treat (NNT) or the number needed to harm (NNH). These estimates quantify the number of patients that would need to be treated with extended adjuvant tamoxifen to cause a beneficial or detrimental event of interest in one patient who would not otherwise have experienced that event. All statistical tests were two-sided, and statistical significance was defined as p<0.05. No corrections were made for multiple testing. Sensitivity analyses were also conducted for trials with heterogeneous eligibility criteria.

## Results

**Figure 1:**
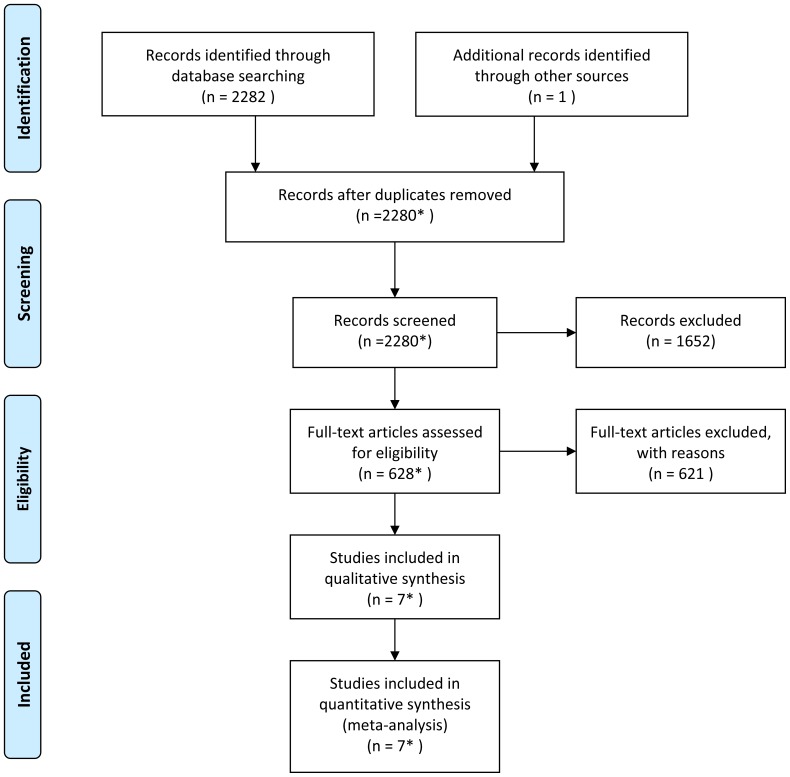
Flow diagram of literature search. *Includes one commentary providing updated efficacy (but no toxicity) results of one of the included randomized trial.

The search identified a total of 2283 citations. After applying our eligibility criteria, 1285 review articles and commentaries and 367 articles of trials conducted in metastatic breast cancer were initially excluded. Of the remaining 630 articles, 621 were excluded as they investigated aromatase inhibitors or chemotherapy in either the control or experimental arms. Three duplicate articles were also excluded as was one study [10] where the control arm was 2–3 years of tamoxifen rather than 5 years. Six citations [5]–[9], [15] reporting results of five studies were therefore eligible for analysis and included two [7], [15] recently reported results of the aTTom trial. A further commentary [16] presenting updated results of one randomized trial [9] was identified through a review of reference lists and was also included in the analysis (see figure 1). Characteristics of included studies are shown in Table 1. Four studies [5], [6], [8], [9] (83%) provided data on disease recurrence for subgroups of patients with ER-positive primary breast cancer (i.e. after excluding ER-negative and ER-unknown cases if applicable); while in one study [7] only data on a combined group of ER-positive and ER-unknown were available. Three [6]–[8] of the five included studies provided follow-up data to 10 years and were included in the assessment of absolute risks.

**Table 1:** Characteristics of Included Studies.

Trial	MedianFollow-up(years)*	Nodepositive(%)	Duration oftherapy incontrol arm(years)	Duration of therapyin experimentalarm(years)	SampleSize	OR forRecurrence	95% CI	*P*
**All Patients**								
ATLAS [8]	7.6¶	40	5	10	12894	0.85	0.76, 0.95	0.006
aTTom [7], [15]	∼9**	31	5	10	6953	0.84	0.74, 0.95	0.006
ECOG E4181/E5181 [9], [16]	9.6	100	5	indefinite	193	0.45	0.23, 0.89	0.02
NSABP B14 [5]	7	0	5	10	1172	1.18	0.80, 1.72	0.41
Scottish Trial [6]	10	23	5	indefinite	342	1.36	0.83, 2.22	0.22
**ER-positive**								
ATLAS [8]	7.6¶	42	5	10	6846	0.84	0.74, 0.94	0.003
aTTom [7], [15]	∼9**	NR	5	10	6953†	0.84	0.74, 0.95	0.006
ECOG E4181/E5181 [9], [16]	9.6	100	5	indefinite	140‡	0.33	0.15, 0.70	0.004
NSABP B14 [5]	7	0	5	10	1172	1.18	0.80, 1.72	0.41
Scottish Trial [6]	10	NR	5	indefinite	132§	0.93	0.46, 1.92	0.85

OR, odds ratio. CI, confidence interval. NR, not reported.

*Median follow-up after randomization.

¶ The mean follow up.

† Data not reported based on ER status, therefore whole cohort analyzed (40% ER-positive, 60% ER-untested).

‡ The reported follow-up for the ER-positive group is shorter than for the whole study population [9] as this subgroup was not reported in the update [16].

§ ER level of <20 fmol/mg on ligand binding assay is usually consistent with negative immunohistochemistry results [44],therefore excluded.

**Estimated follow-up based on time elapsed between two reported results.

### Efficacy

**Figure 2:**
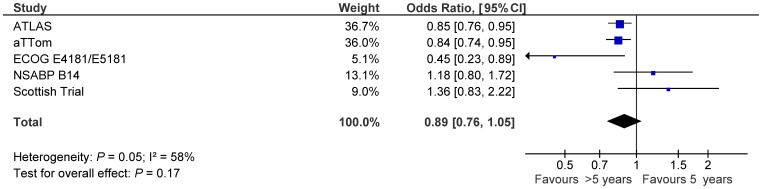
Forest plots of odds ratios for breast cancer recurrence for patients treated with extended adjuvant tamoxifen (>5 years) versus adjuvant tamoxifen (5 years) based on primary analysis of individual trials. Odds ratios for each trial are represented by the **squares**, the **size of the square** represents the weight of the trial in the meta-analysis, and the **horizontal line** crossing the square represents the 95% confidence interval. The **diamonds** represent the estimated pooled effect (labeled total).

Among all randomized patients, extended adjuvant tamoxifen was not associated with a significant reduction in the odds of breast cancer recurrence (OR 0.89, 95% CI 0.76–1.05, p = 0.17, see figure 2).

Subgroup analysis using data from patients with ER-positive primary breast cancer (including one study which also included ER-unknown cases [7]) showed a numerically lower estimate, but did not differ significantly from the whole group (OR 0.85, 95% CI 0.72–1.00, p for difference = 0.66). Exclusion of one study [7] which was unable to differentiate ER-positive and ER-unknown patients failed to show a difference in risk of recurrence (OR 0.89, 95% CI 0.75–1.06, p for difference = 0.60).

**Figure 3:**
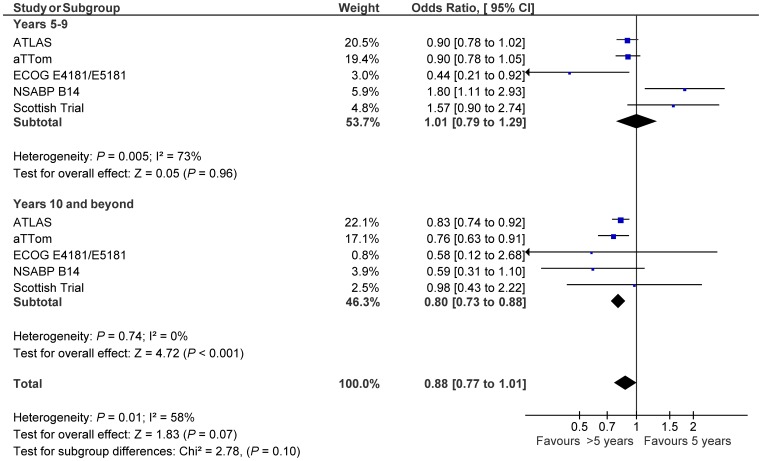
Forest plots of odds ratios for breast cancer recurrence between years 5–9 and in years 10 and beyond for patients treated with extended adjuvant tamoxifen (>5 years) versus adjuvant tamoxifen (5 years) based on primary analysis of individual trials. Odds ratios for each trial are represented by the **squares**, the **size of the square** represents the weight of the trial in the meta-analysis, and the **horizontal line** crossing the square represents the 95% confidence interval. The diamonds represent the estimated pooled effect based for each cohort individually (labeled subtotal) and for all cohorts together (labeled total).

**Figure 4:**
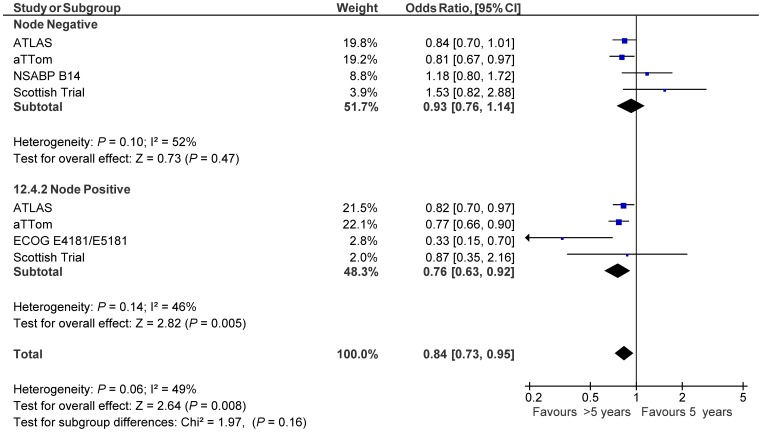
Forest plots of odds ratios for breast cancer recurrence in node negative and in node positive patients with extended adjuvant tamoxifen (>5 years) versus adjuvant tamoxifen (5 years) based on primary analysis of individual trials. Odds ratios for each trial are represented by the **squares**, the **size of the square** represents the weight of the trial in the meta-analysis, and the **horizontal line** crossing the square represents the 95% confidence interval. The diamonds represent the estimated pooled effect based for each cohort individually (labeled subtotal) and for all cohorts together (labeled total).

Data on timing of recurrence were available from all eligible studied. Analysis showed that benefit from extended tamoxifen may occur beyond extended adjuvant therapy (OR 0.80, 95% CI 0.73–0.88) with no evidence of benefit during extended adjuvant therapy (OR 1.01, 95% CI 0.79–1.29). This difference approached, but did not meet statistical significance (subgroup difference p = 0.10, see figure 3). Data based on baseline lymph node status were available from all eligible studies. Analysis showed similar results with apparent benefit seen only among those with lymph node positive disease (OR 0.76, 95% CI 0.63–0.92) and not in those that were lymph node negative (OR 0.93, 95% CI 0.76–1.14, see figure 4). This difference, however, was not statistically significant (subgroup difference p = 0.16). At 10 years of follow-up, the absolute risk reduction was 2.1% in lymph node negative patients (NNT: 49) and 4.1% in lymph node positive patients (NNT: 25). Data on baseline menopause status were available from three studies [6], [8], [9] and among these, 822 women (13%) were documented to be pre-menopausal. There was no apparent effect of menopause status on breast cancer recurrence (OR for pre-menopause 0.80, 95% CI 0.57–1.12 compared with OR for post-menopause 0.86, 95% CI 0.64–1.14, subgroup difference p = 0.76).

Data on distant recurrence were available from three [5], [8],[9] of the five eligible studies. Compared with 5 years of tamoxifen, there was no difference in the odds of distant recurrence with extended adjuvant tamoxifen (OR 0.96, 95% CI 0.66–1.38, p = 0.81). Similarly, there was no association between extended tamoxifen and death after breast cancer recurrence (OR 0.98, 95% CI 0.79–1.21, p = 0.82).

**Figure 5:**
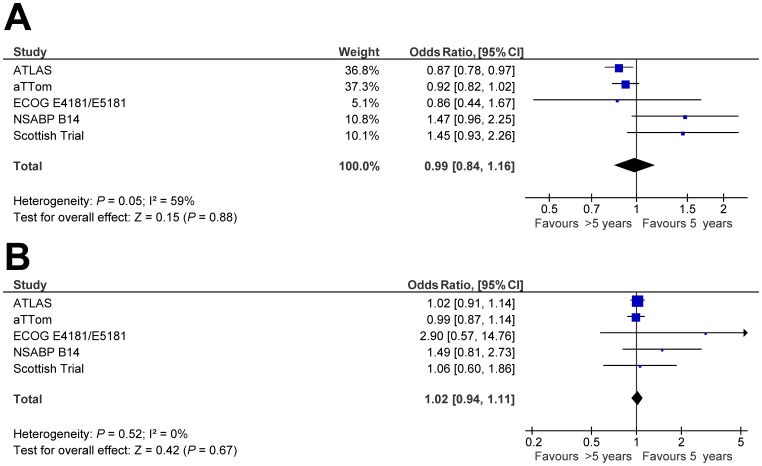
Forest plots of odds ratios for (A) all-cause death and (B) death without recurrence for patients treated with extended adjuvant tamoxifen (>5 years) versus adjuvant tamoxifen (5 years) based on primary analysis of individual trials. Odds ratios for each trial are represented by the **squares**, the **size of the square** represents the weight of the trial in the meta-analysis, and the **horizontal line** crossing the square represents the 95% confidence interval. The diamonds represent the estimated pooled effect based for each cohort individually (labeled subtotal) and for all cohorts together (labeled total).

There was no apparent association between extended tamoxifen and all cause death (OR 0.99, 95% CI 0.84–1.16, p = 0.88, see figure 5A). There was significant heterogeneity among studies (Cochran’s Q p = 0.05 and I^2^ index = 59%). The estimate was sensitive to the modeling method used to weigh studies. Use of fixed effects modeling was associated with a statistically significant and lower OR (p for difference = 0.32), likely as a result of substantially higher weighting for the ATLAS and aTTom trials (36.8% and 37.3% versus 45.7% and 48.2% respectively). Data on all-cause death in those known to have ER-positive disease were available from four studies [5], [6], [8], [9]. Results were similar to those for patients with unselected ER status (OR 1.11, 95 CI 0.79–1.55, p = 0.55). Again significant heterogeneity was seen (Cochran’s Q p = 0.02 and I^2^ index = 69%). Despite, a numerically higher number of deaths in the extended tamoxifen group, there was no association between extended tamoxifen and death without breast cancer recurrence (OR 1.02, 95% CI 0.94–1.11, p = 0.67, see figure 5B).

Data on absolute risk difference and NNT at 10 years of follow-up are shown in Table 2.

**Table 2:** Unweighted absolute risks and number needed to treat (NNT) for events of interest after 10 years of follow-up.

Event of interest	Number ofincluded studies	Absolute risk inextended adjuvanttamoxifen group	Absolute risk inadjuvant tamoxifengroup	Absolute riskdifference	NNT*
Any recurrence (all patients)	3 [6], [8], [15]	12.7%	14.5%	1.8%	57
Any recurrence (excluding ER-negative)	3 [6], [8], [15]	17.5%	20.2%	2.7%	38
Distant recurrence	1 [8]	15.0%	16.2%	1.3%	79
Death after recurrence	3 [6], [8], [15]	7.7%	8.7%	1.0%	101
Death without recurrence	3 [6], [8], [15]	11.9%	11.8%	−0.0008%	−1281
All cause death	3 [6], [8], [15]	15.4%	16.7%	1.3%	79

95% CI, 95% confidence intervals. ER, estrogen receptor.

*Negative value suggests more events in extended adjuvant group (i.e. net harm).

### Safety and Tolerability

Extended adjuvant tamoxifen was associated with a significant increase in endometrial carcinoma (OR 2.06, 95% CI 1.65–2.58, p<0.001). At 10 years of follow-up, the absolute risk increased from 1.1% in the adjuvant tamoxifen group to 2.2% in the extended adjuvant group (NNH 89). Among the small cohort of patients who developed endometrial carcinoma (n = 344), receipt of extended adjuvant tamoxifen was associated with a non-significant lower odds of death from endometrial carcinoma (OR 0.71, 95% CI 0.42–1.20, p = 0.20).

Data on non-breast, non-endometrial cancers were available from four [5], [6], [8], [9] of the five eligible studies. There was no association between extended adjuvant tamoxifen and these cancers (OR 0.91, 95% CI 0.80–1.03, p = 0.14). At 10 years of follow-up, the absolute difference in risk was 0.4% and the NNH was 203.

Extended adjuvant tamoxifen was not associated with a significant reduction in death from cardiovascular disease (OR 0.89, 95% CI 0.73–1.09, p = 0.25). At 10 years of follow-up, the absolute risk changed from 3.2% in the adjuvant tamoxifen group to 2.8% in the extended adjuvant tamoxifen group. The NNT to prevent one cardiovascular death was 236.

Data on venous thromboembolism were only reported in two [8], [9] of the six eligible studies. One study reported the incidence and mortality from pulmonary embolism while the other reported the incidence of deep venous thrombosis. Consequently, these data were not pooled in a meta-analysis.

As only one study was placebo controlled, relative risks of treatment discontinuation could not be assessed. Among those receiving extended adjuvant tamoxifen, treatment was discontinued in 38% of patients (95% CI 37–39%).

## Discussion

Despite a predisposition for late recurrence, long-term endocrine therapy for hormone receptor positive breast cancer is not routinely recommended for all patients [17], [18]. Multiple studies have assessed the hypothesis that extending the duration of endocrine therapy will result in a reduction of breast cancer recurrences. Data are currently available from studies which enrolled patients treated initially with tamoxifen alone and randomized these either to ongoing tamoxifen or AI. Results of these studies have been mixed leading to uncertainty regarding the benefit of extended adjuvant endocrine therapy.

Here we report on a published data meta-analysis of trials of 5 years versus longer duration (more than five years) of tamoxifen. Results show that in unselected patients, extended adjuvant tamoxifen was not associated with a significant reduction in the odds of breast cancer recurrence or a reduction in all cause death. There was significant statistical heterogeneity among included studies and this necessitated the use of random effects modelling. Use of fixed effect modelling or of individual patient data would have led to higher weighting of larger studies such as ATLAS and aTTom and may have led to statistically significant reductions in breast cancer recurrence or all cause death. Such methods were, however, not feasible in the current analysis.

Of interest, among studies reporting a negative association between extended adjuvant tamoxifen and breast cancer recurrence, eligible patients were either all node negative [5] or predominantly (77%) lymph node negative [6]. The remaining three studies [7]–[9], all of which included a greater proportion of lymph node positive patients, individually reported benefit from extended adjuvant tamoxifen. Lymph node involvement appears to be prognostic for late relapse [19], [20]. Furthermore, in women receiving extended adjuvant therapy with AI, lymph node positive disease was also associated with translation of improvements in DFS into improvements in OS [2]. This observation was not seen in lymph node negative patients. Results from the current analysis suggest a trend for more benefit in women with node positive disease.

The association of extended adjuvant tamoxifen and breast cancer recurrence appeared to be influenced by the time of recurrence. Extended adjuvant tamoxifen appeared to have little to no effect on breast cancer recurrence during therapy (years 5 to 9 after initial diagnosis), with a modest reduction in recurrence seen after completion of extended adjuvant therapy (beyond year 10). This finding may be explained by the ongoing effect of the initial (adjuvant) period of tamoxifen therapy. Data from studies of extended adjuvant AI show that despite this well recognized hangover effect [1], patients who discontinue adjuvant tamoxifen as much as 5 years earlier may benefit from extended adjuvant endocrine therapy [21]. These data are of clinical relevance to post-menopausal women treated with tamoxifen alone for the first 5 years. In contrast to our data, a meta-analysis of extended adjuvant AI [22] (after 5 years of adjuvant tamoxifen) showed statistically significant and higher magnitude of reduction of breast cancer recurrence. Furthermore, extended adjuvant therapy with AI was associated with benefit even during the period of treatment [2]. In the absence of comparative data of extended tamoxifen versus extended AI, the earlier benefit seen from AI suggests that in post-menopausal women, provision of extended adjuvant AI may be more favourable than further tamoxifen. Our subgroup analysis supports the potential benefit of extended adjuvant tamoxifen for selected pre-menopausal women. However, pre-menopausal women comprised only a small proportion of all randomized patients (13%) and therefore, there is some uncertainty regarding the potential benefit of extended adjuvant tamoxifen in this group. Extended tamoxifen may also be suitable for women who are intolerant of extended adjuvant AI and those living in areas were AI are prohibitively expensive. The utility of extended adjuvant endocrine therapy in patients who have received either upfront adjuvant AI or a sequencing strategy remains unknown and will be answered by a number of ongoing studies [23]–[25].

Compliance with tamoxifen was suboptimal with 38% of patients discontinuing study drug before completion of 5 years of extended therapy. This rate is slightly higher than that seen in the adjuvant trials where between 20 and 25% of patients discontinued tamoxifen within the first 5 years of treatment [1], [26]. However, this rate is similar to the rate of discontinuation of adjuvant tamoxifen observed in population-based studies [27]. The rate of discontinuation of extended adjuvant AI in the NCIC CTG MA.17 trial was lower, where ∼20% of patients discontinued the extended adjuvant AI and in women age 70 years or older the rate of discontinuation was similar to the placebo [28]. This low level of compliance may underestimate the true efficacy of extended adjuvant therapy.

The estimates of benefit of extended adjuvant tamoxifen could also be affected by the inclusion of patients with ER-negative or ER-unknown cancers. While it was possible to perform a sensitivity analysis after exclusion of patients with ER-negative from all studies and ER-unknown from the ATLAS and the Scottish trial, it was not possible to exclude patients with ER-unknown from the aTTom trial because such data were not reported. The percentage of ER-unknown after exclusion of ER-negative from all studies and ER-unknown from the ATLAS and the Scottish trial was 27%. Furthermore, it was estimated that only 80% of ER-unknown patients in the aTTom trial were ER-positive. The effect of inclusion of these patients is difficult to estimate but may underestimate the efficacy of extended adjuvant tamoxifen.

The effects of extended adjuvant tamoxifen on non-breast cancer deaths are of interest, especially with cardiovascular disease now being the predominant cause of death in older women with breast cancer [29], [30]. Overall, there was no significant difference in the odds of death without recurrence between extended adjuvant tamoxifen and control groups; however, there was a non-significant numerical reduction in the odds of cardiovascular death with extended adjuvant tamoxifen. In postmenopausal women, tamoxifen can have beneficial effects on cholesterol and other serum lipids such as low-density lipoproteins [31], [32]. However, data also show an association between tamoxifen and unfavorable effects on serum triglycerides [33], [34]. This balance between improvements in serum cholesterol and detrimental effect on serum triglycerides may explain the non-significant effects on cardiovascular death in our analysis.

As expected, extended adjuvant tamoxifen was associated with an increase in the incidence of endometrial carcinoma. The proportion of patients with prior hysterectomy in included studies was not uniformly reported, therefore, the estimates of risk for this adverse event in women with an intact uterus are subject to some uncertainty. Interestingly, in our analysis it appeared that endometrial carcinoma which developed in women on extended tamoxifen was associated with lower odds of death than that of those who only received adjuvant tamoxifen. However, the number of analyzable events was small leading to some uncertainty regarding this estimate. The reasons for this observation are not clear, and reports describing the prognosis of tamoxifen-induced endometrial carcinoma have been quite varied [35]–[41]. This finding may be explained by endometrial carcinoma in the extended tamoxifen arm having more a favourable prognosis and consequently a lower risk of death. Alternatively, as included studies were predominantly open-label, it is possible that a more rigorous follow-up and investigation of atypical vaginal bleeding in women receiving tamoxifen compared to controls may have led to earlier detection of endometrial carcinoma.

This study has several limitations. First, this is a meta-analysis of the literature rather than of individual patient data and is based on reports of trials with different durations of follow-up. We were therefore unable to report actuarial rates of recurrence or toxicity. Second, collection of data for the purpose of this meta-analysis was dependent on the rigorous collection and reporting of events of interest by the investigators. It is known that such reporting can be suboptimal, especially for disease specific outcome [42] and for toxicities [43]. Third, information on potentially confounding baseline host factors (e.g. obesity, co-morbidity, family history of events of interest or germline mutation carriage) or the use of concurrent medications which may interfere with tamoxifen action was not available. The effect of this on our results cannot be quantified. Finally, the reported duration of follow-up of some of the included studies was relatively short (less than 10 years). For a disease with a prolonged risk of recurrence, data at longer follow-up (over 10 years) would be more informative.

In conclusion, in unselected patients, extended adjuvant tamoxifen is not associated with a significant reduction in recurrence or a reduction in all-cause death. Patients with lymph node positive breast cancer may derive some reduction in recurrence although the long-term effects of this on all-cause death remain unclear. Individual patient meta-analysis should address whether improvements in the risk of recurrence translate into survival improvements in patients with node positive disease.

## Supporting Information

Checklist S1 PRISMA Checklist.
(DOC)
